# ReportFlow: an application for EEG visualization and reporting using cloud platform

**DOI:** 10.1186/s12911-020-01369-7

**Published:** 2021-01-06

**Authors:** S. Bertuccio, G. Tardiolo, F. M. Giambò, G. Giuffrè, R. Muratore, C. Settimo, A. Raffa, S. Rigano, A. Bramanti, N. Muscarà, M. C. De Cola

**Affiliations:** IRCCS Centro Neurolesi “Bonino Pulejo”, S.S. 113, Contrada Casazza, 98124 Messina, Italy

**Keywords:** Cloud, Public key, Security, Privacy, Role-based access control, Medical reports, Data sharing

## Abstract

**Background:**

The cloud is a promising resource for data sharing and computing. It can optimize several legacy processes involving different units of a company or more companies. Recently, cloud technology applications are spreading out in the healthcare setting as well, allowing to cut down costs for physical infrastructures and staff movements. In a public environment the main challenge is to guarantee the patients’ data protection. We describe a cloud-based system, named ReportFlow, developed with the aim to improve the process of reporting and delivering electroencephalograms.

**Methods:**

We illustrate the functioning of this application through a use-case scenario occurring in an Italian hospital, and describe the corresponding key encryption and key management used for data security guarantee. We used the X^2^ test or the unpaired Student *t* test to perform pre-post comparisons of some indexes, in order to evaluate significant changes after the application of ReportFlow.

**Results:**

The results obtained through the use of ReportFlow show a reduction of the time for exam reporting (*t* = 19.94; *p* < 0.001) and for its delivering (*t* = 14.95; *p* < 0.001), as well as an increase of the number of neurophysiologic examinations performed (about 20%), guaranteeing data integrity and security. Moreover, 68% of exam reports were delivered completely digitally.

**Conclusions:**

The application resulted to be an optimal solution to optimize the legacy process adopted in this scenario. The comparative pre-post analysis showed promising preliminary results of performance. Future directions will be the creation and release of certificates automatically.

## Background

Electroencephalography (EEG) is an electrophysiological monitoring method used to record the electrical activity of the brain in millisecond-range temporal resolution [1]. EEG is used to diagnose neurological disorders such as sleep disorders, epilepsy, dementia, to name a few [2–5]. Differently from other techniques such as computed tomography, positron emission tomography or magnetic resonance imaging, EEG is non-invasive, cheap and fast of using, and this makes it a valuable screening tool although its lower spatial resolution [6]. As several prediction data mining techniques used to support healthcare decision-making [7], EEG signal processing integrated with computational algorithms based on machine learning methods may contribute to a deeper comprehension of the disease, as well as to support physicians in early diagnosis [8]. However, a visual inspection performed by trained experts remains the most reliable manner to diagnose a disease.

The recent advancements of Information and Communication Technologies (ICT) allow accessing a large amount of information remotely [9], and assessing missing value from data as well [10]. In this scenario, the cloud represents a practical solution to the problems of storing and sharing a large amount of electronic health records (EHR) or other types of health data, providing several benefits to the user and organization. Indeed, cloud systems can be used to share information in real-time and to optimize economic and temporal resources [11], besides to be a tool to solve the problem of prediction from a huge healthcare database [12], cutting down costs required by management and administration of physical infrastructures. It allows for higher productivity compared to previous manual exchange of data. However, some security requirements for data sharing in cloud computing systems have to be guaranteed [13]. Thus, the provider must ensure data security and privacy of sensitive information, especially in complex domains like healthcare [14].

According to their accessibility, the cloud systems can be classified into: (1) public (external), when it is accessible from anywhere over the internet such as Google’s Drive online storage; (2) private (internal), when its infrastructure is developed to manage private cloud environment; (3) hybrid, as a combination between public and private cloud systems [15].

The use of a security system based on Public Key Infrastructure (PKI), symmetric and asymmetric key encryption and digital signature may guarantee a fine-grained access control scheme able to maintain the security and integrity of data on public cloud infrastructures, preserving patient privacy, and preventing the policy enforcers from comprehending the access control policies transmitted with the data [16]. Symmetric-key encryption is based on cryptography algorithms using only one key for both encryptions of plaintext (human-readable data) and decryption of the unintelligible ciphertext [17]. On the contrary, asymmetrical encryption uses a pair of public and private keys to encrypt and decrypt the data, according to the RSA algorithm developed by Rivest, Shamir, and Adleman [18]. Thus, the data can be encrypted using the public key and decrypted through the private key, which is known only to the owner. Similarly, digital signature employs asymmetric cryptography to provide data authentication and integrity [19]. Therefore, the private key is used to encrypt owner information data, whereas the public key to check the owner of the data.

In this paper we report a portable PC application, called ReportFlow, that we developed to share sensitive data over public cloud, and to speed and simplify the medical report process of EEGs reporting and delivering. The paper is structured as follows: in the methods section, we introduce the case study that inspired our work and describe the functioning of ReportFlow, proving cloud specifics and the key management adopted. In the next sections, we report and discuss some preliminary results. Finally, we expose the limitations, advantages, and disadvantages of such an application, comparing it with the most recent related works.

## Methods

### Case study

The IRCCS Centro Neurolesi Bonino Pulejo of Messina (Italy) includes a neurological main center and three external facilities where different units take place. EEGs and evoked potentials of children are performed at the neurophysiology diagnostic unit (NDU) placed at the main center, but to report the examination it is needed to wait for a child neuropsychiatrist who comes from the peripheral unit (which is distant about 25 km). Sometimes, this issue can also require a couple of days because of the duties at the peripheral unit.

To fulfil the request of EEGs reporting by optimizing times and costs, we developed, in Python programming language (version 3.7), ad-hoc PC application called ReportFlow for sharing instrumental examinations among members of a clinical team including staff from different units (i.e. child neuropsychiatry, neurophysiology laboratory, and administrative office). ReportFlow exploits the public cloud platform and a PKI system. We tested the application to improve the procedure of EEGs reporting adopted in our hospital setting, which encompasses the four steps summarized in Table 1.

**Table 1:** The EEGs recording and reporting process pre-post the development of ReportFlow

	Before ReportFlow	After ReportFlow
Step 1	At the main centre, the neurophysiology technician performs the EEGs. The recordings are stored on the lab’s PC	At the main centre, the neurophysiology technician performs the EEGs. The recordings are encoded and stored on the Cloud by ReportFlow, i.e. the application updates the synchronized folder containing the EEG records. Then, ReportFlow notifies to the physician that a new examination has been recorded
Step 2	The physician (i.e. the child neuropsychiatrist) bi-weekly goes to the main centre to report the EEGs performed. Then the reports are printed and personally signed by the physician	The physician uses ReportFlow from a PC of any IRCCS facility, selects the EEG record and reports it. The report is encoded and digitally signed by the physician. ReportFlow takes care to notify and update the synchronized folder containing the EEG reports. ReportFlow notifies to the medical clerk that a new examination has been reported
Step 3	EEG reports are archived by a medical clerk	The medical clerk uses ReportFlow to validate the correct archiving of the EEG reports. ReportFlow sends to the patient the EEG report by email
Step 4	The medical clerk calls the patient to warn that the EEG report is ready and can be either personally picked up	If the patient does not provide an email, the medical clerk calls him to warn that the EEG report is ready and can be either personally picked up

### The ReportFlow application functioning

**Fig. 1:**
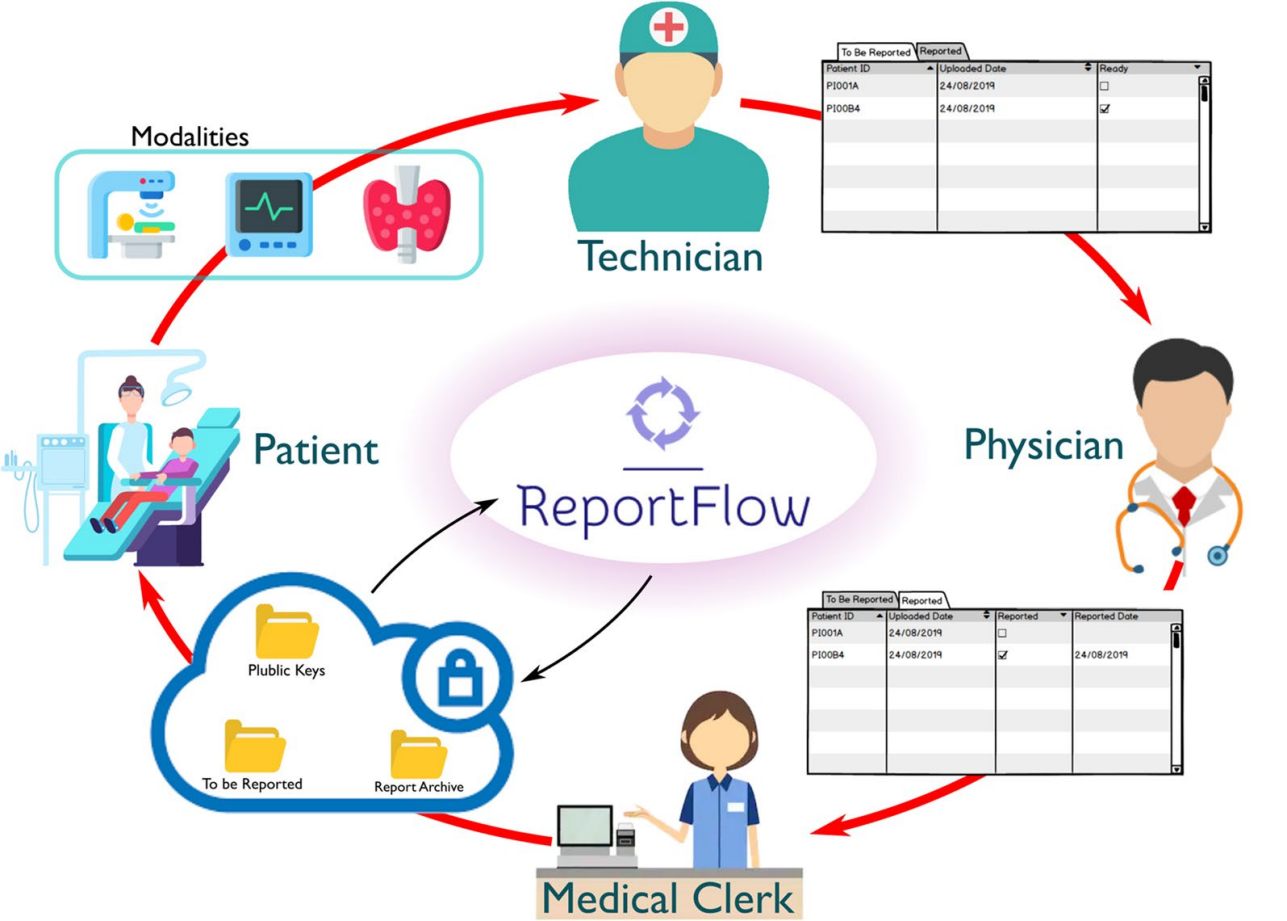
The EEG reporting and delivering process

**Fig. 2:**
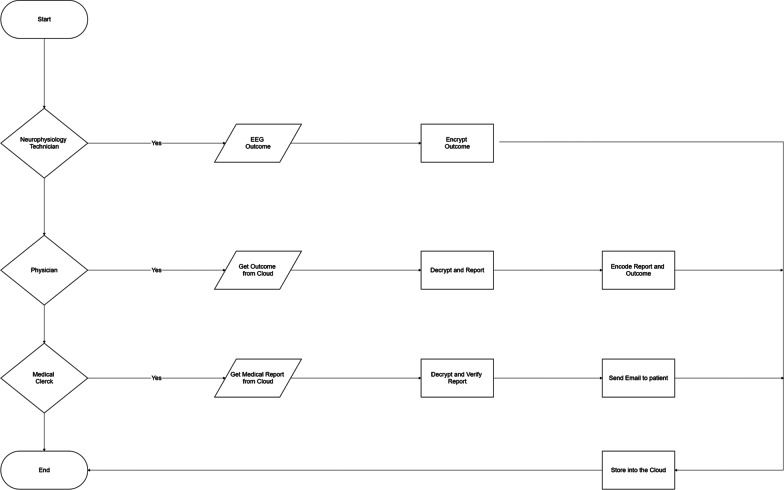
Flow chart of the EEG reporting and delivering process

The functioning of ReportFlow uses a role-based access control (RBAC) [20], an approach to restricting system access to authorized users. Thus, some operations and features are differentiated to the user according to his role, identified by the certificate stored in a USB Write Once Read Many pen drive. Notably, to protect the data stored on the Cloud, ReportFlow takes care of all cryptographic activities, besides to manage all certificates and check their validity by using OpenSSL, an open source general-purpose cryptography library. Public keys and other information are directly stored on the public cloud into specific folders. ReportFlow encrypts the data through a Triple Data Encryption Symmetric Algorithm (Triple DES or 3DES) [21], using a 128 characters randomly generated password before uploading it on the Cloud. This password will be used to decrypt the file when the user (i.e. physician or the medical clerk) will need to use it. ReportFlow encrypts the random password using the public keys of the user according to his role. This ensures that only authorized operators can decode files. The encryption process takes place locally on the PC of the user, whereas the results are uploaded on the Cloud. The cloud syncing activity is not performed directly by ReportFlow but is demanded to the cloud application installed on the operator PC. Figures 1 and 2 show the ReportFlow functioning, whereas an additional text file shows the pseudo-code (see Additional file 1).

### The cloud platform

The cloud platform used in this study is Google Drive online storage of G-Suite [22]. The provider’s account (i.e. the IRCCS), which has unlimited storage space, is used to set and manage a shared drive with employees. The shared drive contains three different folders, which are accessible to employees according to their responsibilities (roles) within the company, e.g. all team members of the NDU. The first folder, shared in read-only mode, includes all certificates; the second folder, shared in read/write mode, includes the EEGs recorded (i.e. XML files containing both the EHR and the diagnostic examination, for each patient); and the third folder shared only to physicians and administrative staff, includes the EEGs reported. To keep in sync, the local folder with Cloud folder the Google Drive File Stream application was used, running on Windows and Mac OSX, while the Google Drive Ocamlfuse was used on Linux OS.

### The cryptography and key generation

For the management and administration of keys and certificates, the software XCA is used. A private key is created for the provider, and with this key, in turn, a Certification Authority (CA) certificate is associated with the NDU involved. Thus, for each subject in NDU, an RSA private key and an X.509 certificate, signed with NDU-CA, are generated. To make the release of multiple certificates easier, the certificate signing request (CSR) is used. CSR is a special message for a CA used to apply for a digital identity certificate [23]. Indeed, CSR contains the public keys of the NDU and some identifying information, i.e. name, working unit, role. The same CSR can be used for different CAs to get several certificates, each of them associated with a different unit, in order to correctly identify the staff member and his associated privileges.

Each member of the NDU is provided with a USB Key containing the duple {private key; X.509 certificate}, as well as the ReportFlow application with needed libraries, and the Cloud tools.

### Data collection

Data referred to two consecutive semesters of 2019 were extracted from the administrative hospital database. To identify the examinations, we selected healthcare services with code 89.14.x (electroencephalogram) and 84.15.x (evoked potential) of the Italian national nomenclature of outpatient specialist care, adopted following the Ministerial Decree of January 2017. The only exclusion criterion applied was to be over 18 years of age.

### Statistical analysis

We defined a list of indexes and we compared them before and after the ReportFlow development, i.e. we compared the first semester of ReportFlow use (T1) with the previous semester (T0). Statistical analysis was performed by using the 3.5.0 version of the open-source software R. A *p* < 0.05 was considered as statistically significant. Results for continuous variables were expressed in mean ± standard deviation, whereas categorical variables in frequencies and percentages. The X^2^ test with continuity correction was used to assess for statistical differences in proportions, whereas the unpaired Student t-test was used to compare continuous variables.

## Results

**Fig. 3:**
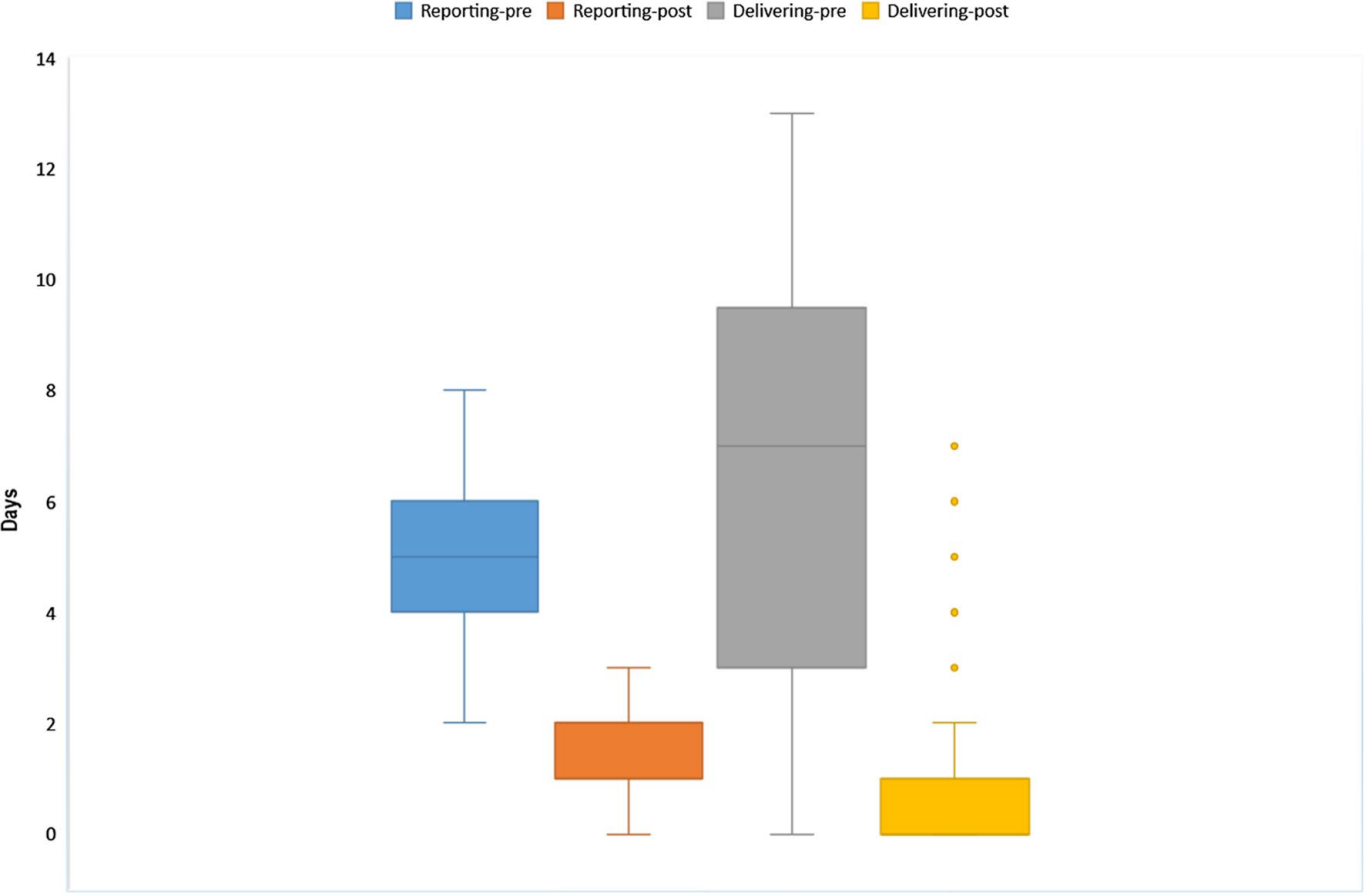
Boxplots of reporting times and delivering times before and after the use of ReportFlow

As reported in Table 2, we found a significant reduction of average times in both EEG exam reporting (*t* = 19.94; *p* < 0.001) and delivering (*t* = 14.95; *p* < 0.001) when the ReportFlow application was used. Figure 3 shows the magnitude of these time reductions, more evident in EEG report delivery times. Moreover, the rate of phone calls to patients was significantly lower (χ^2^ = 94.87; *p* < 0.001), the number of EEG/EP exams performed increase of 20%, and the child neuropsychiatrist was able to visit about 30% of outpatients more than before. Finally, with the introduction of ReportFlow, about 68% of exam reports were delivered completely digitally.

**Table 2:** Pre–post comparisons

Index	T0	T1	*p* value
Number of exams performed	160	192	–
Average time of exam reporting (days)	4.96 ± 1.08	1.23 ± 0.55	< 0.001
Average time of exam report delivering (days)	6.68 ± 3,92	0.69 ± 1.47	< 0.001
Number of exams with dematerialized reporting	0 (0%)	130 (67.7%)	< 0.001
Number of phone calls to patients to notify that the report is ready for being picked up	160 (100%)	75 (39.1%)	< 0.001
Number of outpatient visits by the child neuropsychiatrist	414	538	–

Continuous variables were expressed as mean ± standard deviation, whereas categorical variables as frequencies and percentages

## Discussion

The results confirm that the use of ReportFlow can improve the process of EEG reporting and delivering, reducing times and increasing performance. Indeed, we observed an increase in the number of neurophysiologic examinations performed, as well as the number of outpatients visits at the child neuropsychiatric unit. The service with the greater benefit was the report delivery, thanks to the speedup of the administrative procedure. Therefore, the use of ReportFlow supported the hospital in cost-saving (e.g. for paper, stationery, phone calls) and facilitated the patients as well.

Expanding the use of ReportFlow also to other repositories, in future it will be possible to manage different kinds of data, and offer a wide range of services through a process completely dematerialized, i.e. reporting and delivering of Magnetic Resonance Imaging evaluations, genetic testing and so on.

All crypto functions (coding and decoding) were implemented on the client-side by ReportFlow, without exposing sensitive data over the public cloud. Indeed, ReportFlow uses the OpenSSL PKI system to store data over the public cloud, ensuring the security level needed by healthcare institutions, and also making the EEG reporting process faster and simpler than the process originally used in our structure. Healthcare companies often keep legacy systems, i.e. old method, technology and application programs. Even if these systems work satisfactorily, they could be replaced by more secure, accurate and modern systems [24]. In our case study, the legacy process forced personnel to be physically present at the same location where the neurophysiological exam was carried out, besides requiring printing the EEG reports, which now are directly delivered to the patients digitally by ReportFlow. Thus, the hospital provides a more efficient service by reducing costs and waiting times for both personnel and patients. Indeed, physicians can report an evaluation everywhere and at any free time, e.g. during a break between two visits; the medical clerk can monitor in real-time the state of the reporting, soliciting the physician to report urgent cases; the patient has to wait less time for obtaining the exam report. On the whole, all involved staff can monitor the process through the ReportFlow interface, which includes a notification system.

## Limitations, advantages and disadvantages

The main limitation of this study concerns the key management, since the key and certificate preparation is not automated and needs trained SysAdmins. Thus, in future we plan to develop an internal tool for USB key preparation with the generation of private key and CSR, allowing the staff members to request the certificate automatically as well. Moreover, the application does not check the quality control of EEGs and the management of report format. However, in future it will also include a semi-automated data quality control script before data sharing, in order to ensure that planned acquisition parameters and data annotation are followed and data files are not corrupted. A disadvantage of this method could be the possibility of occurring in conflicting activities, for example when two or more physicians simultaneously report the same outcome. In order to minimize such a conflictual eventuality, Report Flow application applies some techniques i.e. using lock file (with double check) and deleting from the cloud all decoding passwords except for the current user. Unfortunately, this problem could not be totally solved since the cloud infrastructure is here used as a global notification mechanism.

Working in a public environment, the most important disadvantage of ReportFlow concerns data security. Although the protection and privacy of data hosted by cloud providers are not so high as in a private cloud, ReportFlow encode data using a random generated key in turn encoded using a PKI system and, with this cryptography process, the security of the patients’ sensitive data can be protected from third parties. Moreover, even if we have used Google Drive, ReportFlow is not linked to any specific cloud services and it is economically convenient. Indeed, ReportFlow did not constitute an additional cost for the company because Google is the actual Cloud Service, which is one of the most stable and scalable cloud solutions. Finally, ReportFlow does not need specific customization, but it is only based on syncing features of the cloud service. This avoids limits and restrictions in customization due to the public infrastructure of the cloud.

## Related work

The advent of the cloud has created a new paradigm in providing and using infrastructures, leading to new challenges concerning security and privacy of sensitive data, especially in the healthcare environment [25]. Several contributions in method development for healthcare data sharing we can find in literature. In this section we summarized some previous methods of data sharing on cloud in the healthcare environment, and compared them with ReportFlow according to the access control, the encryption technique, and the key management (Table 3).

**Table 3:** Comparison among data sharing methods in the healthcare environment, taking into consideration the adopted methodology, aim, could accessibility, access control, and encryption technique

First author	Method	Setting	Cloud accessibility	Access control	Encryption technique
Bertuccio	ReportFlow	EEGs data sharing for medical reporting	Public	RBAC	CP-ABE
Pugazhenthi	ID HKE	Securing and sharing of health data	Public	Not indicated	SE-KAC
Ahmed	CONNECT and ad hoc protocol	EHRs data sharing	Public	DAC	Not indicated
Basu	Fusion	EHRs data sharing and managing	Public and private	Not indicated	Symmetric
Marwan	Shamir’s secret share scheme	Securing health data	Public and private	Shamir’s secret share scheme	Shamir’s secret share scheme
Sneha	K-Anonymity	Securing and sharing of EHRs	Public	Not indicated	Re-encryption
Hwang	–	Data securing	Public	Not indicated	Knapsack public-key cryptosystem
Wei	EFADS	Data sharing for outsourcing data	Not indicated	Not indicated	Proxy re-encryption
Chu	–	Securing and sharing of scalable data	Public and private	Shamir’s secret share scheme	Key-aggregate encryption
Rezaeibagha	–	Securing and sharing of EHRs	Public and private	RBAC	Cryptographic building blocks for secret sharing
Thilakanathan	Ad hoc Java application	Securing and sharing of ECG data	Public	Not indicated	Public-key cryptography system
Tran	–	Securing and sharing of cloud-based social networks data	Public	Personal secret key to encrypt and decrypt data	Proxy re-encryption

*RBAC* role-based access control, *DAC* discretionary access control, *SE-KAC* scalable and enhanced key-aggregate cryptosystem, *CP-ABE* Ciphertext-policy attribute-based encryption

Recently, Pugazhenthi and Chitra [26] described a method called IDHKE able to securely share the secret keys to the receiver in the stage of decryption, since an encryption is used. The key is safely generated using one random prime number, a master secret key and parameter value. In their work, Ahmed et al. [27] described a method providing patient privacy and accountability in the health information sharing environment by using the open-source CONNECT software to enable eHealth Exchange specifications, although their research lacked a thorough representation of dynamic access-control policy solutions. In another study, Basu et al. presented a cloud-based platform for securely managing and sharing healthcare information at a large scale, but without an access structure to clarify data-sharing management [28]. Similarly, Marwan et al. [29] proposed a novel methodology based on a multi-cloud concept, to meet the security level required by health care, avoiding loss of data, unauthorized access, and privacy disclosure. Sneha and Asha [30] proposed to use k-anonymity for privacy-preserving EHR. Ibrahim et al. [31] provided a comprehensive solution for EHR by using a cryptographic role-based technique and the Kerberos protocol to carry session keys. However, these studies require neither a privacy-aware cloud infrastructure or specific protocols, with the disadvantage of having to sustain hardware acquisition costs, besides requiring skilled human resources to manage, monitor and support the cloud infrastructure. This can be a problem for healthcare companies, which are lacking software developers and computer scientists, and they usually pay for cloud services such as Google or Microsoft Azure. Therefore, ReportFlow was aimed to offer a working model simple and efficient, without using special protocols or middleware applications over the cloud infrastructure.

Works in public key encryption environments are very limited. For example, Hwang et al. [32] constructed a knapsack encryption scheme for the problem of public key encryption based on permutation combination algorithm. However, this permutation method appeared to be not efficient to the security of the scheme [33]. Shao et al. proposed a public key encryption protocol supporting multiple receivers for medical information sharing based on bilinear maps. Data owner stored only one copy of his encrypted file and its corresponding encrypted keywords on the cloud for multiple designated receivers [34]. A data-sharing system was proposed by Wei et al. [35], in which the data holder encrypts data with the public key and then uploads it to the cloud servers, regardless of various access requirements. Chu et al. [36] presented a public-key encryption scheme that produces constant-size cypher-texts for efficient delegation of decryption rights in the cloud data sharing in a hierarchical structure. However, these works in public key encryption have long ciphertext related to the number of receivers, do not support receiver revocation without re-encrypting, and do not preserve the membership of receivers. All crypto functions (encryption and decryption) of ReportFlow were implemented on the client-side, without exposing sensitive data over the public cloud.

Most papers about EHR access control in public key environments are based on attribute-based encryption (ABE) to encrypt data and to provide the hierarchical access structure for fine-grained data sharing. However, in a practical application, EHR data could be stored in multiple clouds due to the need for scalability and privacy. Rezaeibagha and Mu [37] proposed an EHR data sharing system, based on several cryptographic building blocks and secret sharing, with RBAC to protect patient’s privacy stored in different types of clouds (i.e. private and public clouds), whereas ReportFlow is based on Ciphertext-policy attribute-based encryption (CP-ABE) scheme, one of the most suitable in a public environment since it can guarantee data owners’ direct control over their data and provide a fine-grained access control service. Indeed, users obtain their private keys only after data encryption, without knowledge of the actual set of users that will be able to decrypt only by specifying the actual policy [38].

Concerning electrophysiological data sharing, Thilakanathan et al. [39] presented a system using a sensor connected to a mobile phone via Bluetooth to stream encrypted electrocardiographic (ECG) data to the cloud. According to Tran et al. [40], the system is based on proxy re-encryption where keys are partitioned and shared with other physicians. Revoking a user would simply involve removing the corresponding physician’s key partition in the cloud. Our system is similar but all encryption processes are handled directly by ReportFlow and not require other external devices, except for the one that contains the certificates. This device can be a USB key or a smartcard or also a Bluetooth connected mobile phone.

## Conclusions

The ReportFlow application developed for sharing, visualizing, reporting and delivering EEG records, resulted to be an optimal solution to optimize the legacy process adopted in our hospital. Report Flow provides a user-friendly graphical interface in order to have a good learning curve for the hospital staff. Using ReportFlow the reporting process becomes independent by the location: technicians can take the diagnostic examination everywhere, also in patients’ homes using a portable EEG recorder, and the physician can visualize and evaluate the EEG tracing at any time, even from a remote location. Moreover, the EEG report is instantly available, and the administrative staff can archive it in real-time, while the application automatically delivers it to the patient. The comparative pre-post analysis showed promising preliminary results of performance, although the application is still in the testing phase. Notably, the report delivering service was sensitively speeded up due to the improvement of the whole process.

Health data security is the main purpose of Report Flow. Such purpose is achieved executing all encoding and decoding processes locally exploiting the OpenSSL PKI system. Thus, the public cloud is used only as a storage of encrypted data.

Future directions will be the creation and release of certificates automatically, as well as the implementation of an automatic process for revocations management. A future challenge will be to improve the current locking mechanism, to avoid simultaneity in data transcription, and notify early the progress of the reporting process.

## Supplementary Information

**Additional file 1**. Pseudo-code of the NetworkFlow application according to the role of the user.

## Data Availability

The data that support the findings of this study are available from IRCCS Centro Neurolesi Bonino Pulejo, but restrictions apply to the availability of these data, which were used under license for the current study, and so are not publicly available. Data are however available from the authors upon reasonable request and with permission of IRCCS Centro Neurolesi Bonino Pulejo.

## Abbreviations

EEG: Electroencephalography
PKI: Public Key Infrastructure
EHR: Electronic health records
NDU: Neurophysiology diagnostic unit
ABE: Attribute-based encryption
CA: Certification Authority
3DES: Triple Data Encryption Standard
DES: Data Encryption Standard
GUI: Graphical User Interface
